# The Bond-Slip Relationship at FRP-to-Brick Interfaces under Dynamic Loading

**DOI:** 10.3390/ma14030545

**Published:** 2021-01-23

**Authors:** Di Zhang, Jun Yang, Li Yuan Chi

**Affiliations:** State Key Laboratory of Explosion Science and Technology, Beijing Institute of Technology, Beijing 100081, China; dizhang@bit.edu.cn (D.Z.); yangj@bit.edu.cn (J.Y.)

**Keywords:** FRP, brick, bond–slip, dynamic loading, slip rate

## Abstract

Interface debonding between fiber reinforced polymers (FPR) and substrates is the principal failure mode for FRP-reinforced structure. To understand the bond–slip relationship at FRP-to-brick interfaces under dynamic loading, the influences of the dynamic enhancement of material performance on the bond–slip curve were studied. Single-lap shear tests under two different loading rates were performed, and the slip distribution curves at different loading stages were fitted to derive the bond–slip relationship. Then a numerical model considering the strain rate effects on materials was built and verified with test results. Further, the influences of brick strength, FRP stiffness and slip rate on the bond–slip relationship were investigated numerically. The research results show that FRP stiffness mainly influences the shape of the bond–slip curve, while brick strength mainly influences the amplitude of the bond–slip curve. The variations of the bond–slip relationship under dynamic loading, i.e., under different slip rates, are mainly caused by the dynamic enhancement of brick strength, and also by the dynamic enhancement of FRP stiffness, especially within a specific slip rate range. The proposed empirical formula considering dynamic FRP stiffness and dynamic brick strength can be used to predict the bond–slip relationship at the FRP-to-brick interface under dynamic loading.

## 1. Introduction

Externally bonded fiber reinforced polymer (FRP) is widely used as a proven reinforcement technique for masonry structures [1,2,3,4,5]. Practical applications and numerous studies have found that the debonding of FRP from the surface of substrates (e.g., concrete, clay brick and mortar) is the principal failure mode of reinforced structures [1,6,7,8,9]. The bond–slip relationship between FRP and substrate is the basis for understanding the process of interfacial debonding. The masonry structure is likely to be subjected to dynamic loads such as earthquakes, explosions and impacts. Experimental studies of the FRP-to-substrate interface show that the dynamic interfacial strength is significantly higher than that of static [10,11,12,13], and the dynamic bond–slip relationship is naturally different from that of static [14,15,16,17,18]. An accurate bond–slip relationship can be used for strength calculations of FRP-reinforced structures, helping to design safer and more economical FRP-reinforced solutions. Therefore, the study of the bond–slip relationship at the FRP-to-brick interface under dynamic loading is of great significance.

Almost all the methods for deriving the bond–slip relationship at the FRP-to-substrate interface are based on the pull test [19] (including the single-lap shear test, the double-lap shear test and the beam test) or its numerical simulation. According to the differences in the test data used, the methods for calculating the bond–slip relationship are roughly divided into three: one direct method and two indirect methods.

The direct method is most commonly used. The method directly applies the differential and integral of the axial strain of the FRP to calculate the bond stress and slip, respectively, and then derive the bond–slip relationship by synthesizing the data from different loading stages [20]. Based on the direct method of using strain data, different models have been established to fit the bond–slip relationship. These bond–slip models all conform to the tendency of tensile softening, but with different shapes: (1) bilinear [21,22], e.g., Monti et al.’s model [22]; (2) multilinear [23,24], e.g., Ghiassi et al.’s model [24]; (3) single curve type [25,26,27,28,29,30,31], e.g., Popovics et al.’s model [25] and its development of Nakaba et al.’s model [26] and Savioa et al.’s model [27]; (4) double curve type, e.g., Dai and Ueda’s model [32] and Lu et al.’s model [33]; (5) mixed type with line and curve, e.g., Pan and Wu’s model [34]. All of these five types of models appear in the study of the bond–slip relationship at the FRP-concrete interface.

Some studies have suggested that there are three main reasons why a consistent, reliable and accurate bond–slip relationship cannot be derived with the direct method. First, the debonding process is difficult to capture due to the highly nonlinear and brittle nature of local fractures [35]. Second, the irregularities in the surface treatment and adhesive layer cause an irregular fluctuation in strain measurements [36]. Third, the random distribution of cracks and aggregates in concrete also induces irregular fluctuations in strain measurements, resulting in a drastic variation in bond stress calculated from strain data [33]. To overcome this issue, Ueda and Dai et al. [35,37,38] ignored the dense strain information, fitted the load–slip curve at the loading end and then derived the bond–slip relationship indirectly based on a simple and rigorous analytical method. To further eliminate the chance errors of the experimental measurements and improve the accuracy of the analysis results, Wu et al. [36,39,40] fitted the slip distribution curves at different loading stages simultaneously to calculate the bond–slip relationship indirectly.

Summarizing the concerns of the above references, it can be found that the research on the bond–slip relationship is mainly focused on the FRP-to-concrete interface, while there is less research on the FRP-to-brick interface. The research on the bond–slip relationship at the FRP-to-brick interface under dynamic loading is even more limited. Besides, due to the harsh requirements of the dynamic loading control on the experimental equipment, most of the studies are on the tensile strength of the interface and lack the study of the bond–slip relationship.

Accordingly, the bond–slip relationship at the FRP-to-brick interface under dynamic loading is investigated in this paper. Firstly, a numerical model of the FRP-to-brick interface was constructed based on the single-lap shear tests under two different loading rates. Secondly, the effects of FRP stiffness and brick strength on the bond–slip relationship were studied numerically. Finally, the dynamic enhancement effect on material performance was integrated to study and analyze the dynamic bond–slip relationship under different slip rates.

## 2. Single-Lap Shear Tests

The digital image correlation (DIC) method was adopted to implement full-field strain measurements, and a special fixture was designed to ensure stability during the dynamic loading. The CFRP-to-brick (CFRP, carbon fiber reinforced polymer) single-lap shear tests under two different loading rates were performed on a universal testing machine.

### 2.1. Materials and Specimen Preparation

The bricks used in the tests are standard commercial clay bricks with a size of 240 × 115 × 53 mm^3^. The carbon cloth is a unidirectional woven carbon fiber fabric UT30-30G produced by Toray Japan. The epoxy adhesive HM-180C3P is a two-component fiber adhesive produced by Horse China specifically for structural reinforcement. Their specific material parameters are shown in Table 1.

**Figure 1:**
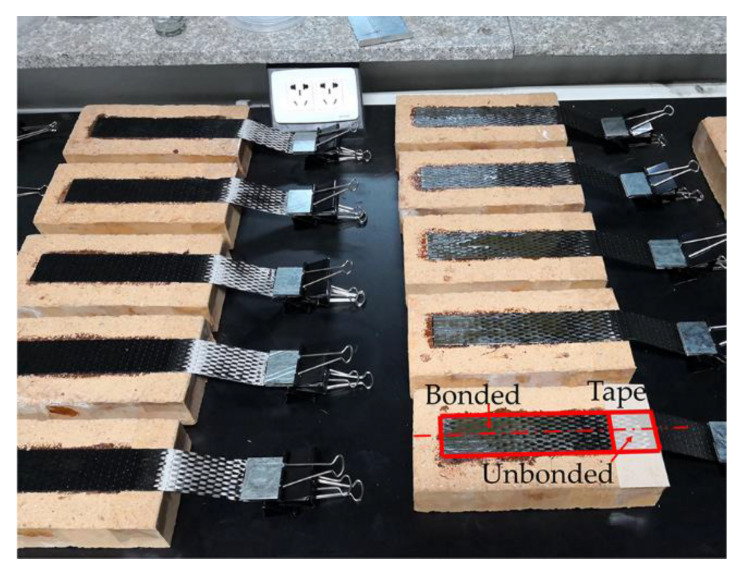
Carbon fiber reinforced polymer (CFRP)-to-brick specimens.

The CFRP-to-brick specimens are shown in Figure 1. The carbon cloth with a width of 40 mm was bonded on the centerline of the brick, which contains a 160 mm long bonded region and a 40 mm long unbonded region. The surface of the bricks was sanded smooth and cleaned previously. Then the unbonded region was covered with tape to prevent impregnation by the adhesive. Finally, the carbon cloth was bonded to the brick by the wet lay-up method.

### 2.2. Instruments and Test Procedures

**Figure 2:**
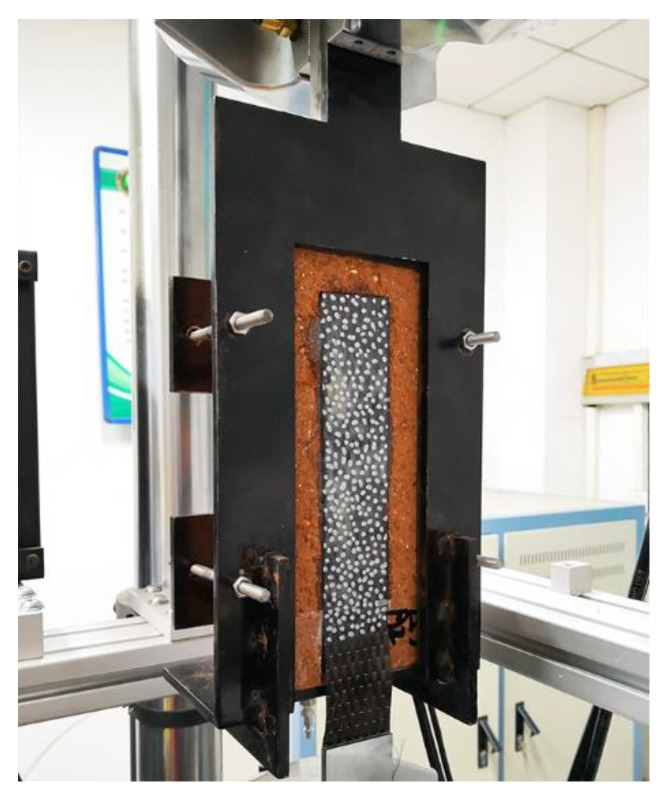
Fixture for single-lap shear tests.

With the fixture as shown in Figure 2, the single-lap shear tests were performed on an MTS universal testing machine (MTS, Eden Prairie, MN, America). The loading rate of the MTS universal testing machine with an ultimate load of 100 KN ranges from 1 to 1000 mm/min. Two groups of tests were performed, Group A: 10 mm/min and Group B: 1000 mm/min. Each group contains five specimens.

**Figure 3:**
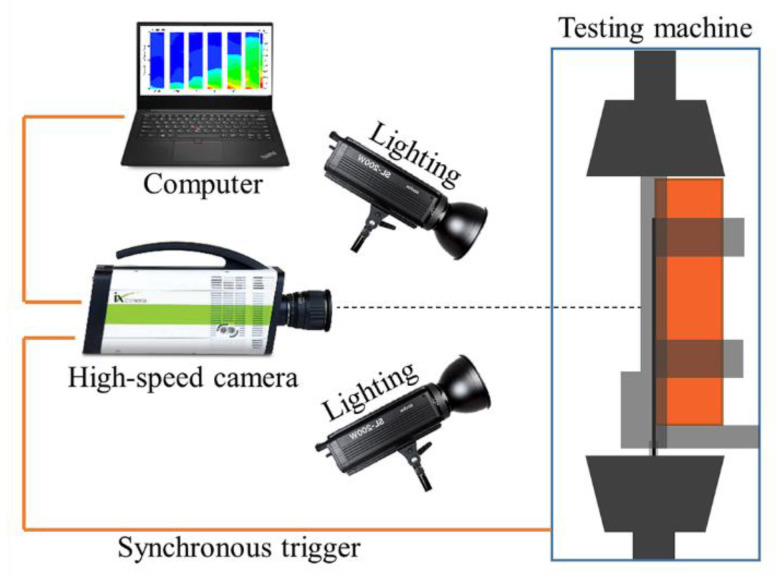
The strain measurement system by digital image correlation (DIC).

The strain was measured by a two-dimensional digital image correlation (DIC) method combined with a high-speed camera (iX Cameras, Rochford Essex, UK). As shown in Figure 3, the debonding process was recorded by a high-speed camera (ix cameras i-SPEED716) with a maximum frame rate of 500,000 fps. The required light was provided by two 200 W LED lights. The high-speed camera was triggered by a TTL (Transistor-Transistor Logic) pulse from the testing machine.

### 2.3. Test Results and Analysis

**Figure 4:**
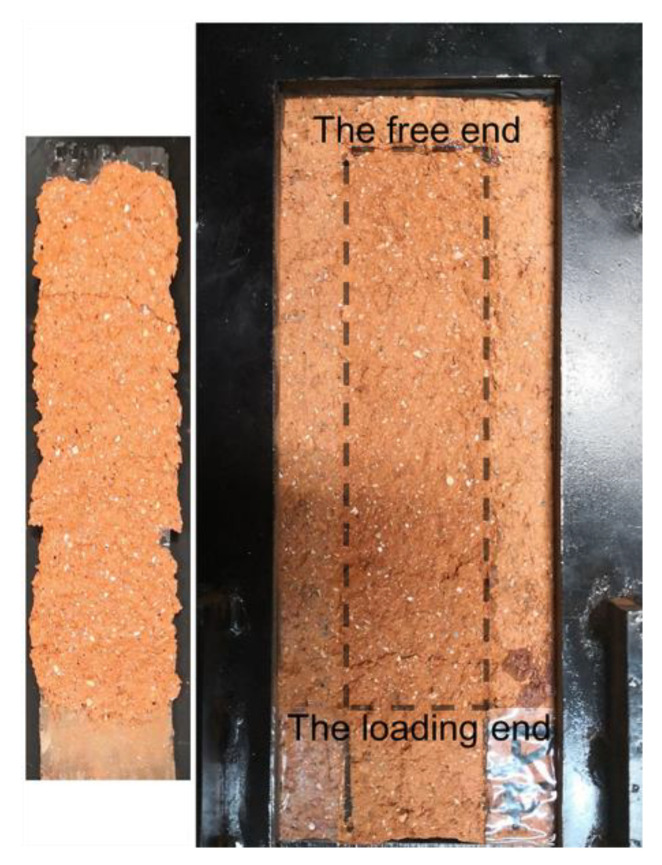
The peeling of CFRP and a thin layer of brick.

The test results revealed that all specimens exhibited the peeling of CFRP and a thin layer of brick, as shown in Figure 4, the fracture of the brick dominated the failure of FRP-to-brick interface. The test results are in agreement with the related literature [30,41].

**Figure 5:**
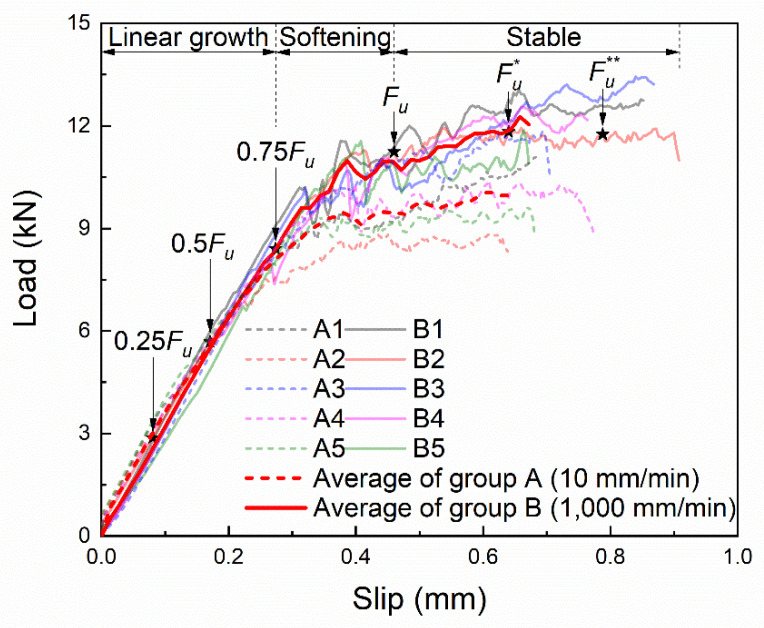
Load–slip curves of the tests.

The load–slip curves at the loading end are shown in Figure 5. The loading process can be roughly divided into three stages: linear growth stage, softening stage and stable stage. With the increase of slip, the tensile force first goes through the linear growth stage and the softening stage to reach the ultimate load *F_u_*, then into the stable stage. In the stable stage, the slip further increases, the tensile force remains basically unchanged.

Comparing the average of the two groups of curves, it can be found that the linear growth rate of both groups is the same, but the linear growth stage of group B is longer so that the ultimate load is larger, which is 1.2 times of that of group A. Besides, Group B also has a longer stable stage and ends up with a larger slip.

**Figure 6:**
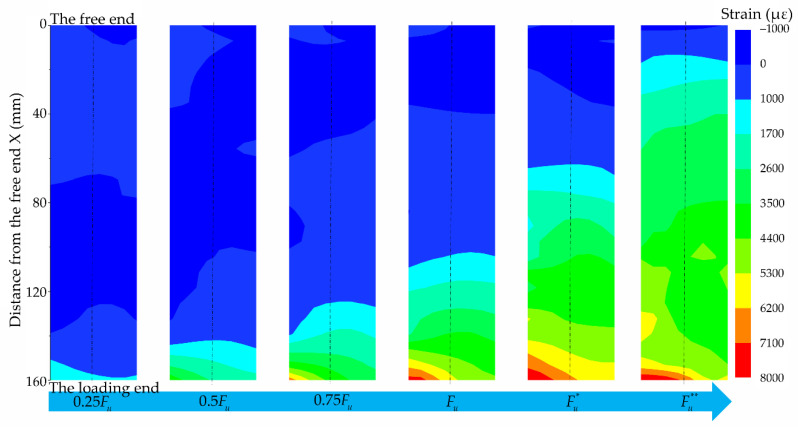
The strain contours of specimen B2.

To deeply understand the process of interfacial failure, the strain at different loading stages marked in Figure 5 was analyzed. The corresponding strain contours obtained by the DIC method are shown in Figure 6, in which the process of strain develops from the loading end to the free end can be clearly seen. Before the ultimate load *F_u_*, the strain increases while it develops from the loading end to the free end. After the ultimate load *F_u_*, the strain continues to develop towards the free end while the strain at the loading end hardly increases, indicating that the interface at the loading end may have been damaged.

**Figure 7:**
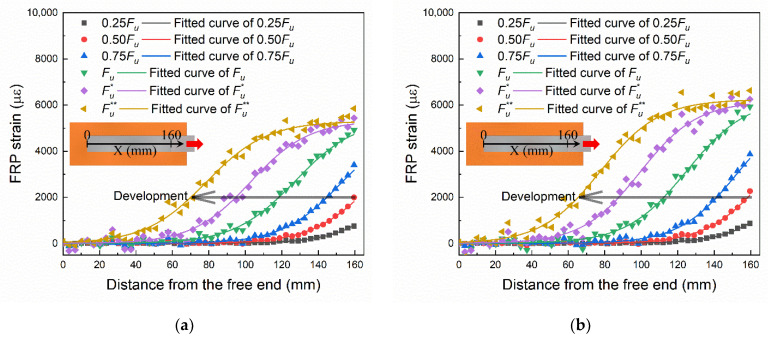
The strain distribution data and the fitted curves of: (**a**) specimen A4 (10 mm/min); (**b**) specimen B2 (1000 mm/min).

The strain on the median line marked in Figure 6 was extracted. The strain distribution data and the fitted curves of specimen A4 and B2 are shown in Figure 7a,b, respectively. The strain development at the loading end (X = 160 mm) matches their load–slip relationships shown in Figure 5. The development of strain from the loading end to the free end can be clearly seen in Figure 7. By comparing Figure 7a,b, it can be found that the final strain of B2 (1000 mm/min) is larger than that of A4 (10 mm/min). There are irregular fluctuations in strain measurements, as shown in Figure 7. It is because of the irregularities in the surface treatment and the random distribution of cracks and aggregates in brick. The formula for fitting the curves in the figure will be given in the next section along with the derivation of the bond–slip relationship.

### 2.4. Bond–Slip Relationship

**Figure 8:**
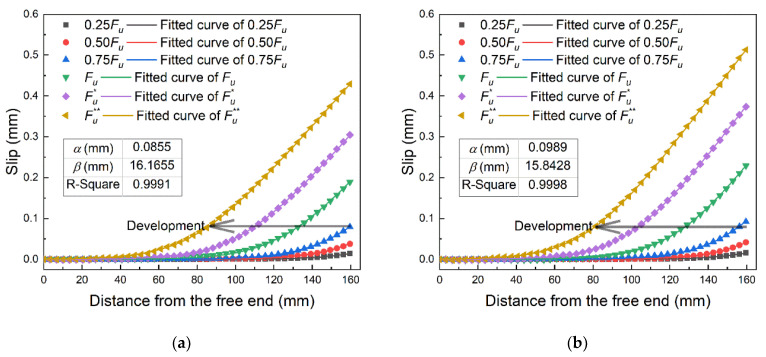
The slip distribution data and the fitted curves of: (**a**) Specimen A4 (10 mm/min); (**b**) Specimen B2 (1000 mm/min).

The slip distribution data derived by integrating the strain is shown in Figure 8. To eliminate the chance errors of the experimental measurements and improve the accuracy of the analysis results, the method proposed by Wu et al. [36,39,40] was applied to calculate the bond–slip relationship. The following mathematical function was used to fit the slip distribution curves at different loading stages simultaneously:

(1) $$s(x)=\alpha \ln(1+e^{(x-x_{0})/\beta })$$

where *s* is the slip, *x* is the distance from the free end, *α* and *β* are the shared fitting parameters and *x*_0_ is the fitting parameter that varies with different loading stages. The fitted results are shown in Figure 8.

The strain distribution can be derived from the slip distribution [36]:

(2) $$\varepsilon (x)=\frac{ds(x)}{dx}=\frac{\alpha }{\beta (1+e^{-(x-x_{0})/\beta })}$$

Then, the distribution of the bond stress can be obtained as follows [36]:

(3) $$\tau (x)=E_{f}t_{f}\frac{d\varepsilon (x)}{dx}=E_{f}t_{f}\frac{\alpha }{\beta^{2}}\frac{e^{-(x-x_{0})/\beta }}{{\left(1+e^{-(x-x_{0})/\beta }\right)}^{2}}$$

where $E_{f}t_{f}$ is called the FRP stiffness, $E_{f}$ is the FRP modulus of elasticity and $t_{f}$ is the FRP thickness.

Equation (1) can be expressed as [36]:

(4) $$e^{(x-x_{0})/\beta }=e^{s(x)/\alpha }-1$$

Substituting Equation (4) into Equation (3) yields the bond–slip relationship [36]:

(5) $$\tau (s)=E_{f}t_{f}\frac{\alpha }{\beta^{2}}e^{-s/\alpha }(1-e^{-s/\alpha })$$

Further, parameters such as the maximum bond stress $\tau_{\max}$, the local slip $s_{0}$ at the maximum bond stress and the interfacial fracture energy $G_{f}$ can be derived [40]:

(6) $$\{\begin{array}{c} \tau_{\max}=\frac{E_{f}t_{f}\alpha }{4\beta^{2}}\\ s_{0}=\alpha \ln2\\ G_{f}=\frac{E_{f}t_{f}\alpha^{2}}{2\beta^{2}} \end{array}$$

**Figure 9:**
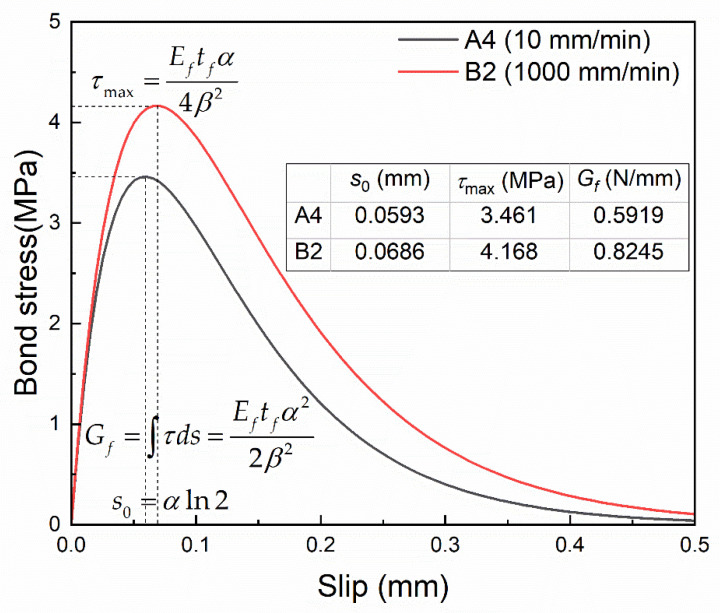
Bond–slip curves for Specimens A4 and B2.

The bond–slip parameters obtained by using the aforementioned method are listed in Table 2, and the bond–slip curves for Specimens A4 and B2 are shown in Figure 9. It can be found that the maximum bond stress and the corresponding slip are greater for Group B (1000 mm/min) compared with Group A (10 mm/min), and the curve coverage area i.e., interfacial fracture energy is greater for Group A as well.

It should be noted that the interfacial fracture energy is the energy consumed for FRP peeling from the substrate per unit area of FRP, not the fracture energy of cracks in the substrate material. The fracture energy of cracks in the substrate material is strain-rate-independent [42,43]. However, the corresponding fracture surface under the FRP is not flat, as shown in Figure 4, and it may change with the loading rate [18]. The variation of the fracture surface may be responsible for the dynamic enhancement of the interface, but the specific microscopic mechanism requires further investigation based on more specimens.

By comparing the load–slip curves, strain distribution, slip distribution and bond–slip curves of group A and group B, it is indicated that the interface behavior of FRP-to-brick has a significant dynamic enhancement effect. This may be related to the strain rate effect on the mechanical performance of materials such as FRP and brick under dynamic loading.

## 3. Numerical Analysis and Validation

To further investigate the bond–slip relationship at the FRP-to-brick interface under various loading rates, a plastic-damage model was established to simulate the deformation and damage of brick in the finite element software ABAQUS. The strain rate effect on the materials was considered, and the debonding process of the FRP-to-brick interface was simulated and compared with the test results.

### 3.1. Numerical Modeling

There are two finite element methods to model the bond behavior: the interface modeling method and the direct modeling method. The method using interface elements (such as CZM, cohesive zone method) is commonly adopted for modeling FRP-reinforce structures due to heavy computational demands. A constitutive law for the interface elements must be obtained previously. This is thus not really a predictive approach for the bond behavior but for structural behavior [44]. The other is the direct modeling method, in which debonding is simulated by modeling the failure of the substrate adjacent to FRP.

As described in Section 2.3, the fracture of the brick dominated the failure of the FRP-to-brick interface. Therefore, the direct modeling method, which assumes a perfect adhesion between FRP and substrate, was used to simulate the FRP-to-brick interface [42,43,44]. The plastic-damage model was used to simulate the deformation and damage of the brick [45]. Thus the slippage of the interface and the damage of the brick are simulated.

**Figure 10:**
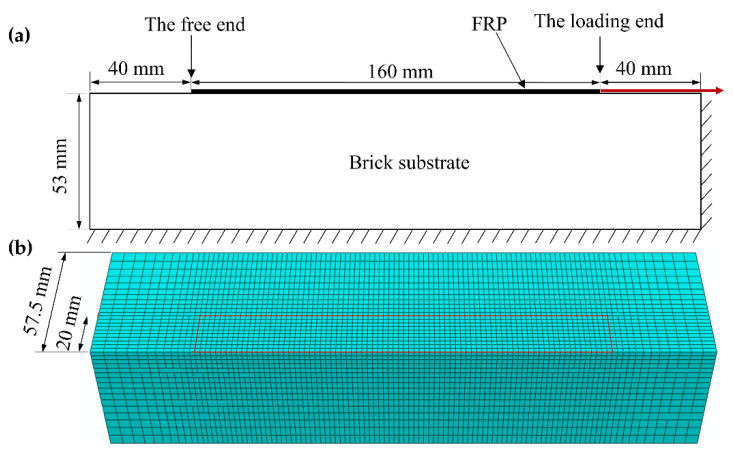
The numerical model of the single-lap shear test: (**a**) boundary conditions; (**b**) mesh.

A three-dimensional numerical model for the single-lap shear test was constructed according to the dimensions of the specimen and the boundary conditions of the test, as shown in Figure 10. Due to the symmetry of the structure and load, only half of the model was built. The brick was modeled with C3D8 eight-node hexahedral elements, and the FRP was modeled with S4R four-node shell element. The FRP and brick near the interface use the same mesh size, and the corresponding nodes were tied separately, so a perfect adhesion between FRP and brick was fulfilled. The bottom and right side faces of the brick were fixed.

The mechanical behavior of FRP was described with a linear elastic model. The mechanical behavior of clay bricks is similar to that of concrete. There is a stiffness degradation caused by the propagation of micro-cracks and micro-defects, and there is a plastic deformation such as slip and flow associated with the mesoscopic mechanism of materials deformation. The correct constitutive relationship of clay bricks should be a plastic-damage constitutive that reflects both damage and plastic deformation.

### 3.2. Plastic-Damage Model

The plastic-damage model in ABAQUS was improved and developed by Lee and Fenves [45] based on Lubliner [46]. The model uses the damage combined with plastic strain to simulate the inelastic behavior of clay bricks. Since the stiffness degradation caused by tensile and compressive plastic strains are considered, this model is suitable for clay bricks under complex loading conditions.

The material failure is achieved by stiffness degradation in the plastic-damage model. Isotropic stiffness degradation is assumed. The concepts of effective stress and strain decomposition are employed. Then the Cauchy stress tensor can be expressed as [45]:

(7) $$\sigma =(1-D)\bar{\sigma }=(1-D)E_{0}(\varepsilon -\varepsilon^{p})$$

where the damage factor $D=1-(1-d_{t})(1-d_{c})$ consists of tensile damage $d_{t}$ and compression damage $d_{c}$, $\bar{\sigma }$ is the effective stress tensor, $E_{0}$ is the initial elastic modulus, ε is the strain tensor and $\varepsilon^{p}$ is the plastic component of the strain tensor.

The nonassociative flow rule is adopted in the model. The plastic potential function associated with effective stress is in the Drucker-Prager form [45]:

(8) $$\Phi (\bar{\sigma })=\sqrt{{\left(\xi f_{t0}\tan\psi \right)}^{2}+3J_{2}(\bar{\sigma })}+\frac{1}{3}I_{1}(\bar{\sigma })\tan\psi$$

where $I_{1}$ is the first invariant of the stress tensor, $J_{2}$ is the second invariant of the stress deviator, $f_{t0}$ is the initial uniaxial tensile strength, *ξ* is the smoothing parameter and ψ is the dilatancy angle. The plastic parameters used in this paper are listed in Table 3, where *f*_b0_/*f*_c0_ and *K* are yield parameters. A detailed explanation of these parameters can be found in the relevant literature [45,47].

The axial compressive strength $f_{c}$ of MU15 clay bricks under quasi-static is 11.4 MPa and the elastic modulus is 7630 MPa. The shape of the compression curve refers to GB50010-2010 [48]. Few experimental studies focused on the tensile properties of clay bricks, the tensile strength (0.25 $f_{c}$) and curve shape; refer to the study of D’Altri et al. [49].

To overcome the mesh sensitivity problem due to the softening behavior and stiffness degradation of the plastic-damage model [47,49], regularization of this model was achieved by scaling the fracture energy with equivalent length. A detailed description of this can be found in the relevant literature [44]. Scaled fracture energy is noted to affect the crack speed in some studies [50] because no damping and no friction is included in the interactions between damaged material points. However, the damage results are not affected by the mesh size [51]. In this paper, the material strength is set according to the loading rate, and the simulation results for the bonding behavior are not influenced by the crack speed.

### 3.3. Strain Rate Effects on Materials

The FRP did not fail during interfacial debonding, so this study only considers the strain rate effect on its elastic modulus. Zhang et al. [52] found that the strain rate effect on the elastic modulus of unidirectional CFRP sheets has obvious stage differences, and the dynamic enhancement factor (DIF) is:

(9) $$DIF_{E_{f}}=\{\begin{array}{cc} 0.0039\log_{10}\dot{\varepsilon }+1.0229 & 1\times 10^{-5}\leq \dot{\varepsilon }\leq 20\;s^{-1}\\ 0.1462\log_{10}\dot{\varepsilon }+0.8378 & 20<\dot{\varepsilon }\leq 160\;s^{-1} \end{array}$$

The compressive strength of bricks is a key parameter that influences interface performance. Zhang et al. [53] carried out the compressive strength tests of clay bricks under different strain rates and obtained the dynamic enhancement factor of compressive strength:

(10) $$DIF_{f_{c}}=\{\begin{array}{cc} 0.114\log_{10}\dot{\varepsilon }+1.567 & 1\times 10^{-5}\leq \dot{\varepsilon }\leq 76\;s^{-1}\\ 1.097\log_{10}\dot{\varepsilon }-0.281 & 76<\dot{\varepsilon }\leq 300\;s^{-1} \end{array}$$

### 3.4. Results Validation and Analysis

#### 3.4.1. Validation with Test Results

**Figure 11:**
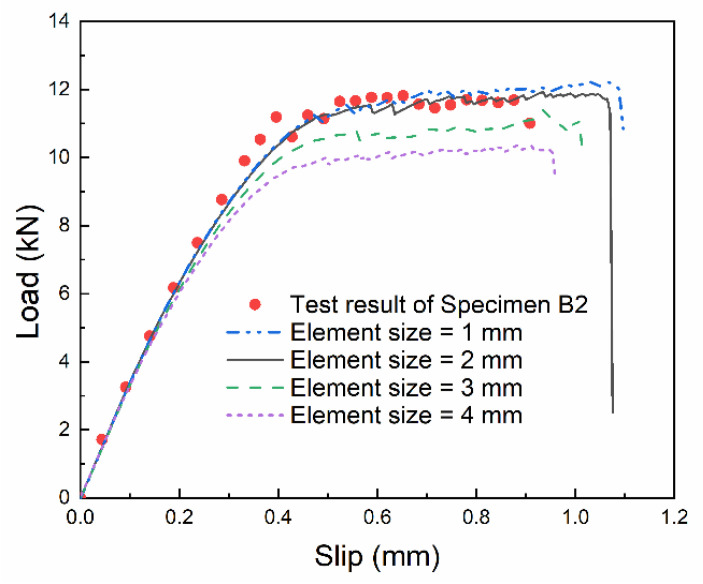
Mesh convergence analysis.

**Figure 12:**
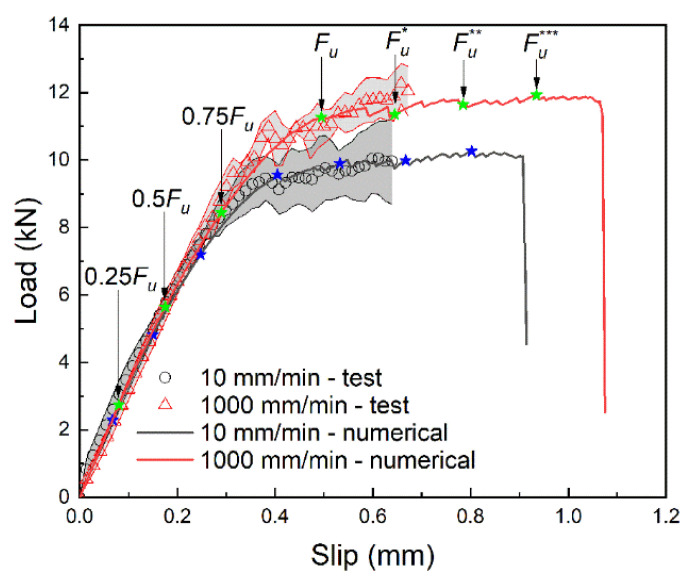
Comparison of load–slip curves between numerical and test.

By comparing the load–slip curves at the loading end simulated with different element sizes, as shown in Figure 11, the mesh convergence was analyzed, and 2 mm was adopted by considering both computational efficiency and accuracy of results. The aforementioned numerical model was used to simulate the single-lap shear tests under the loading rates of 10 mm/min and 1000 mm/min, respectively. The calculated load–slip curves at the loading end were compared with the test results, as shown in Figure 12, where the test results are the mean and standard deviation over the common slip range. It can be found that the linear growth stage and the stable stage of the numerical results are in good agreement with the test results, which proves the reliability of the numerical model.

**Figure 13:**
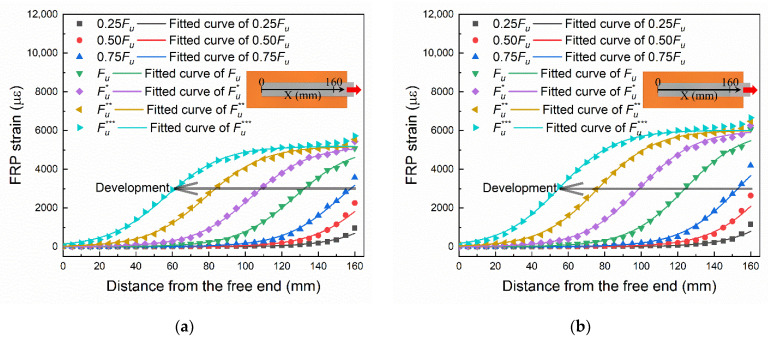

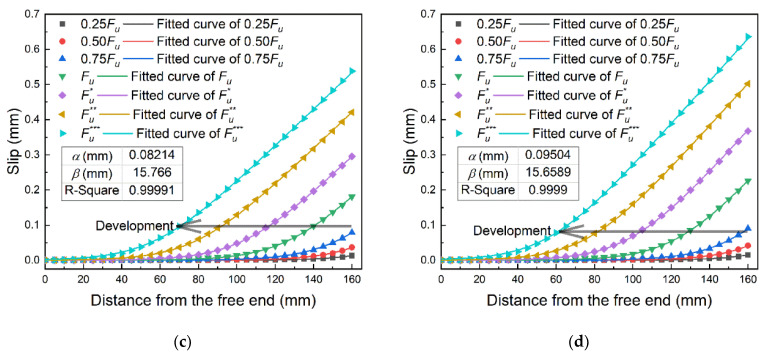
Numerical results of strain and slip distribution: (**a**) strain distribution of 10 mm/min; (**b**) strain distribution of 1000 mm/min; (**c**) slip distribution of 10 mm/min; (**d**) slip distribution of 1000 mm/min.

In the same way as analyzing the test data, firstly, the strain distribution data at different loading stages marked in Figure 12 were extracted, as shown in Figure 13a,b. Then the slip distribution data were obtained by integrating the strain, as shown in Figure 13c,d. Finally, the seven slip distribution curves were fitted by Equation (1), and the fitted parameters *α* and *β* were substituted into Equation (5) to derive the bond–slip relationship.

**Figure 14:**
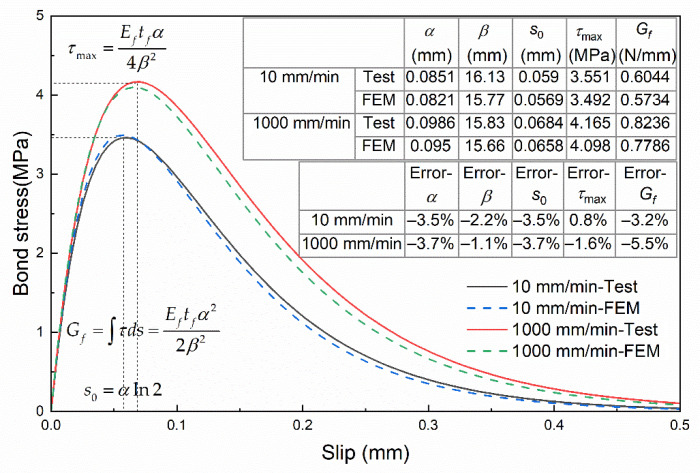
Comparison of bond–slip curves between numerical and test.

The numerical results of the bond–slip curves were compared with the test results, as shown in Figure 14. As the feature parameters of the bond–slip relationship, the errors of the numerical results for $\tau_{\max}$, $s_{0}$ and $G_{f}$ relative to the test results were calculated. The errors under the loading rate of 10 mm/min were −3.5%, 0.8% and −3.2%, respectively; the errors of 1000 mm/min were −3.7%, −1.6% and −5.5%, respectively.

The strain rate effect on the material strength used in this paper is a macroscopic mechanical model. The generation and development of cracks are not simulated. Therefore, the dynamic enhancement of the interfacial fracture energy in the numerical simulations is mainly due to the higher brick strength being considered at higher loading rates.

#### 3.4.2. Debonding Analysis

**Figure 15:**
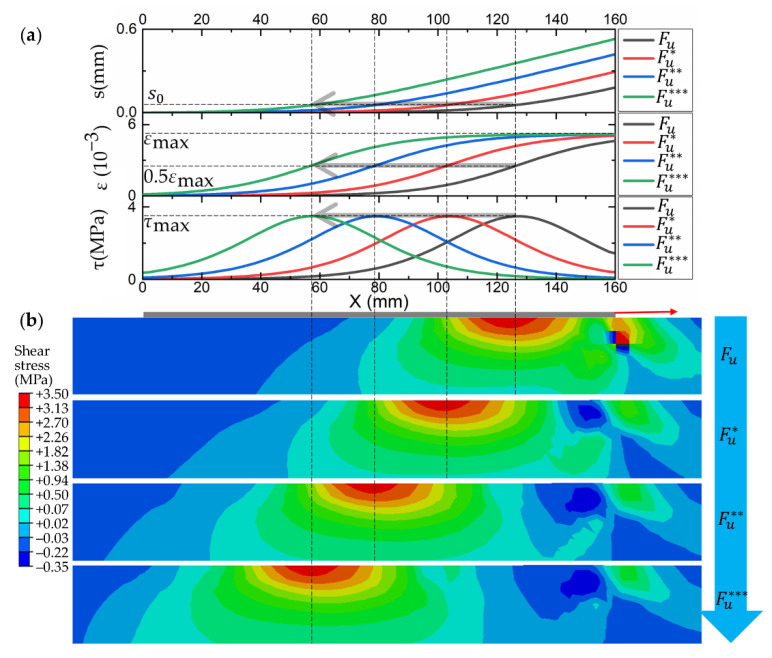
The development of interface debonding: (**a**) the slip distribution, strain distribution and shear bond stress distribution at different loading stages; (**b**) the shear bond stress contours at different loading stages.

The interface debonding process was analyzed by taking a specimen of 10 mm/min as an example. As shown in Figure 15a, the slip distribution, strain distribution and bond stress distribution at different loading stages can be derived by substituting the fitted parameters *α*, *β* and the corresponding $x_{0}$ into Equations (1)–(3), respectively. Overall, with increasing loading, the interfacial debonds from the loading end to the free end. The strain and slip of FRP develop toward the free end, while the interfacial shear bond stress concentration region moves toward the free end. The slip shows an exponentially monotonically increasing distribution during debonding. The shear bond stress shows a similar distribution as Gaussian, which also can be found from the shear bond stress contours shown in Figure 15b. The maximum bond stress in the contours corresponds one-to-one with the peaks in the bond stress distribution, as indicated by the dashed line in Figure 15. In addition, the shear stress concentration caused by squeezing occurs near the loading end, which is the result of the combined effect of boundary conditions and loading conditions.

The numerical results of the load-slip and bond–slip relationships are in good agreement with the test results, and the numerical results give a better understanding of the development of interface debonding. Therefore, the numerical model based on the plastic-damage constitutive and the strain rate effects on materials can simulate the FRP-to-brick interface behavior under different loading rates.

## 4. Parameter Study on Dynamic Behavior

Numerous experimental studies of FRP–substrate interfaces have shown that the FRP stiffness and the substrate strength are the main parameters affecting the bond–slip relationship [20,29,34,38,41,54]. In this section, firstly, the effects of single variables of FRP stiffness and brick strength on the bond–slip relationship at the FRP-to-brick interface were investigated. Then the strain rate effects on FRP stiffness and brick strength were taken into account together to study the bond–slip relationship under different slip rates.

### 4.1. Effect of FRP Stiffness

In the study of the bond–slip relationship, the product of the elastic modulus $E_{f}$ and thickness $t_{f}$ of FRP is generally called the FRP stiffness. The FRP stiffness is mainly determined by FRP type and thickness and may be affected by the loading strain rate. Therefore, different FRPs (CFRP: carbon fiber reinforced polymer, GFRP: glass fiber reinforced polymer and BFRP: basalt fiber reinforced polymer), number of layers (1ply: one layer, 2ply: two layers and 3ply: three layers) and dynamic increase factor (1.2, 1.4, 1.6 and 1.8) were considered in the parameter study. The uniaxial compressive strength of the bricks was set to 11.4 MPa, and the numerical model was used to calculate the bond–slip relationship with different FRP stiffness. The specific calculation settings and results are shown in Table 4. It should be noted that the $\bar{E_{f}t_{f}}$, $\bar{\alpha }$, $\bar{\beta }$, ${\bar{\tau }}_{\max}$, ${\bar{s}}_{0}$ and ${\bar{G}}_{f}$ in Table 4 are the standardized parameters of $E_{f}t_{f}$, α, β, $\tau_{\max}$, $s_{0}$ and $G_{f}$, respectively. Specimen CFRP-1ply was used as a reference, so all the standardized parameters of Specimen CFRP-1ply are 1. The purpose of parameter standardization is to facilitate the observation of parameter sensitivity.

**Figure 16:**
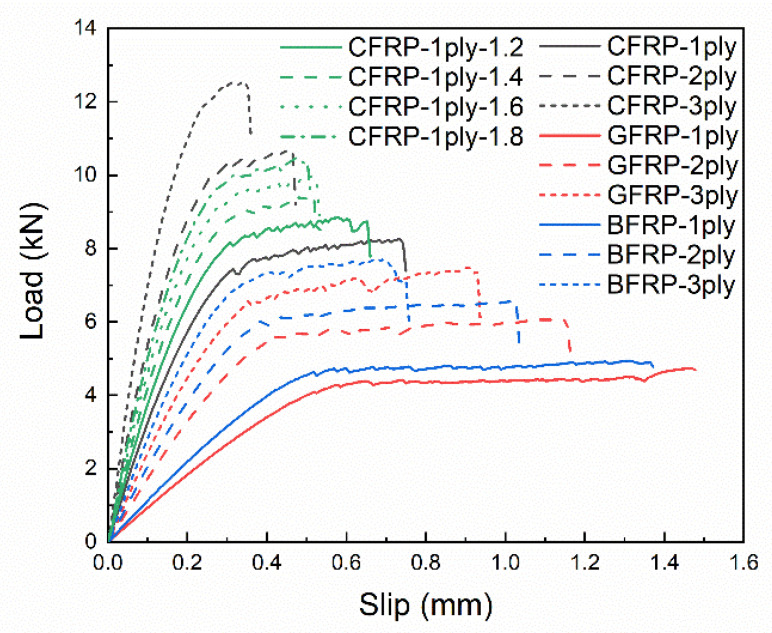
Load-slip curves with different fiber reinforced polymer (FRP) stiffness.

**Figure 17:**
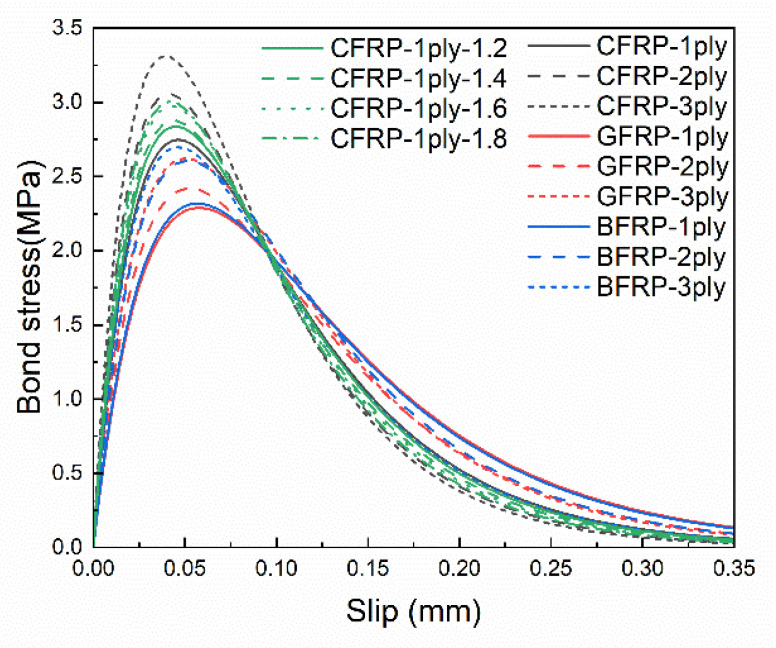
Bond–slip curves with different FRP stiffness.

The load-slip curves with different FRP stiffness are shown in Figure 16. As the FRP stiffness increases, it can be found that the ultimate load increases significantly, while the final slip decreases significantly with no obvious stable stage in CFRP-3ply. This phenomenon can be explained by the two extreme cases of FRP stiffness. By assuming that the FRP stiffness is infinitely large, i.e., the FRP is rigid, the slip at the loading end is identical to the slip at the free end. All the regions in contact with the FRP are simultaneously stressed, so the ultimate load will be large and the final slip will be small. On the contrary, if the FRP stiffness is infinitesimal, the deformation of the FRP at the loading end cannot develop towards the free end. Only the regions contacted by the FRP at the loading end will be stressed, so the ultimate load will be small and the final slip will be large. Moreover, from the point of view of energy conservation, the work done by the tensile force should be equal to the fracture energy of the interface debonding under static loading. If the area of the fracture surface keeps constant, the work done by the tensile force should be fixed, so the ultimate load increases while the final slip decreases. The same phenomenon can be found in the corresponding bond–slip curves, as shown in Figure 17, where the maximum bond stress increases with increasing FRP stiffness while the corresponding slip at the maximum bond stress decreases.

**Figure 18:**
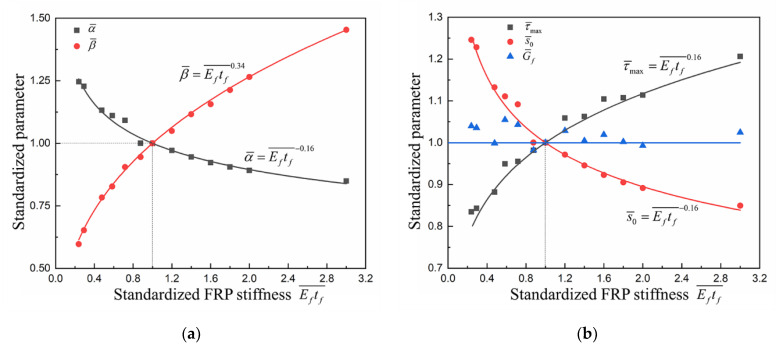
The influences of FRP stiffness on bond–slip parameters: (**a**) *α* and *β*; (**b**) $\tau_{\max}$, $s_{0}$ and $G_{f}$.

A specific understanding of the effects of FRP stiffness on the bond–slip relationship can be found by further quantitative analysis of the bond–slip parameters. The relationships between standardized *α*, *β* and standardized FRP stiffness are shown in Figure 18a. As the FRP stiffness increases, *α* decreases while *β* increases, and both the standardized parameters of *α* and *β* have a power function relationship with the standardized FRP stiffness. The changes in interfacial fracture energy, maximum bond stress and the corresponding slip with FRP stiffness can also be calculated according to Equation (6). As shown in Figure 18b, the changes in maximum bond stress and the corresponding slip have the opposite trend and the same magnitude; thus, the interfacial fracture energy remains unchanged. This is consistent with the analysis of the load–slip curves and bond–slip curves.

Therefore, FRP stiffness mainly influences the shape of the bond–slip curve. The maximum bond stress increases with increasing FRP stiffness, and the corresponding slip decreases accordingly; thus the interfacial fracture energy keeps constant.

### 4.2. Effect of Brick Strength

The brick strength mentioned in this paper refers specifically to the axial compressive strength. The quasi-static strength of MU15 clay bricks is 11.4 MPa, and its strength at the strain rate of 200 s^−1^ is 25.57 MPa calculated by Equation (10). The FRP stiffness is consistent with CFRP-1ply in Table 3 and keeps constant. The bond–slip relationships under nine different brick strength in the range of 11.4 MPa to 25.57 MPa were calculated, the specific calculation settings and results are shown in Table 5.

**Figure 19:**
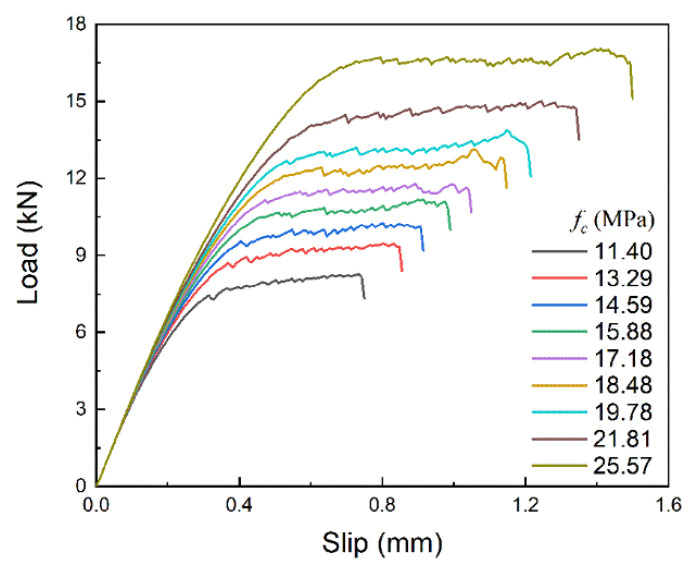
Load-slip curves with different brick strength.

**Figure 20:**
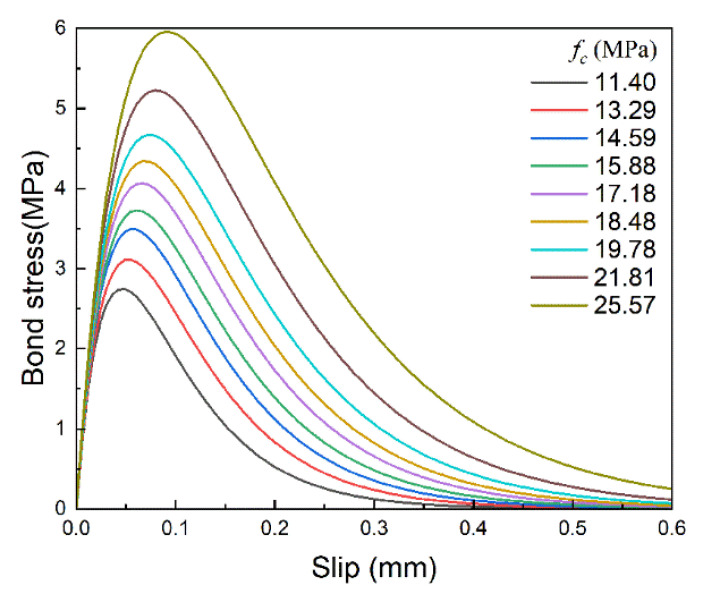
Bond–slip curves with different brick strength.

The load–slip curves with different brick strengths are shown in Figure 19. As the brick strength increases, it can be observed that both the ultimate load and the final slip increase significantly, which indicates that the work done by the tensile force increases. The same phenomenon can be found in the corresponding bond–slip curves, as shown in Figure 20, where both maximum bond stress and the corresponding slip increase with increasing brick strength, making the curve coverage area, i.e., interfacial fracture energy, increase significantly.

**Figure 21:**
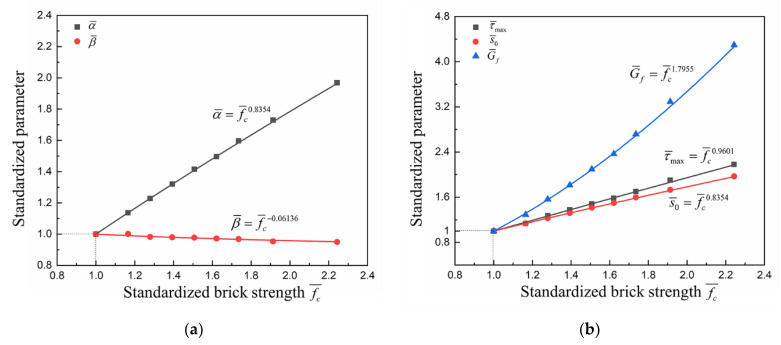
The influences of brick strength on bond–slip parameters: (**a**) *α* and *β*; (**b**) $\tau_{\max}$, $s_{0}$ and $G_{f}$.

The relationships between standardized *α*, *β* and standardized brick strength are shown in Figure 21a. With the increase in brick strength, *α* increases significantly, while *β* decreases slightly, and both the standardized parameters of *α* and *β* have a power function relationship with the standardized brick strength. The changes in interfacial fracture energy, maximum bond stress and the corresponding slip with brick strength can also be calculated according to Equation (6). As shown in Figure 21b, variations of the maximum bond stress and the corresponding slip have a similar trend, which indicates that the shape of the bond–slip curve remains basically unchanged. Therefore, the interfacial fracture energy increases significantly with brick strength.

Being different from FRP stiffness, the brick strength mainly influences the amplitude of the bond–slip curve. Both the maximum bond stress and the corresponding slip increase as the brick strength increases, and the shape of the bond–slip curve remains almost unchanged; thus, the interfacial fracture energy increases significantly.

### 4.3. Effect of Slip Rate

#### 4.3.1. Empirical Formulas and Validation

Based on the effects of FRP stiffness and brick strength on the bond–slip relationship in the above two sections, empirical formulas for *α* and *β* were derived by combining the fit results in Figure 18a and Figure 21a:

(11) $$\{\begin{array}{c} \alpha =0.04778{\left(E_{f}t_{f}\right)}^{-0.16}f_{c}^{0.8354}\\ \beta =0.4954{\left(E_{f}t_{f}\right)}^{0.34}f_{c}^{-0.06136} \end{array}$$

Then the empirical formulas for the maximum bond stress, the corresponding slip at the maximum bond stress, and the interfacial fracture energy were obtained with Equation (6):

(12) $$\{\begin{array}{c} \tau_{\max}=\frac{E_{f}t_{f}\alpha }{4\beta^{2}}=0.04867{\left(E_{f}t_{f}\right)}^{0.16}f_{c}^{0.9601}\\ s_{0}=\alpha \ln2=0.03312{\left(E_{f}t_{f}\right)}^{-0.16}f_{c}^{0.8354}\\ G_{f}=\frac{E_{f}t_{f}\alpha^{2}}{2\beta^{2}}=0.004651f_{c}^{1.7955} \end{array}$$

Where the units of *α*, *β*, $t_{f}$ and $s_{0}$ are mm; the units of, $f_{c}$, $E_{f}$ and $\tau_{\max}$ are MPa; and the unit of $G_{f}$ is N/mm.

**Figure 22:**
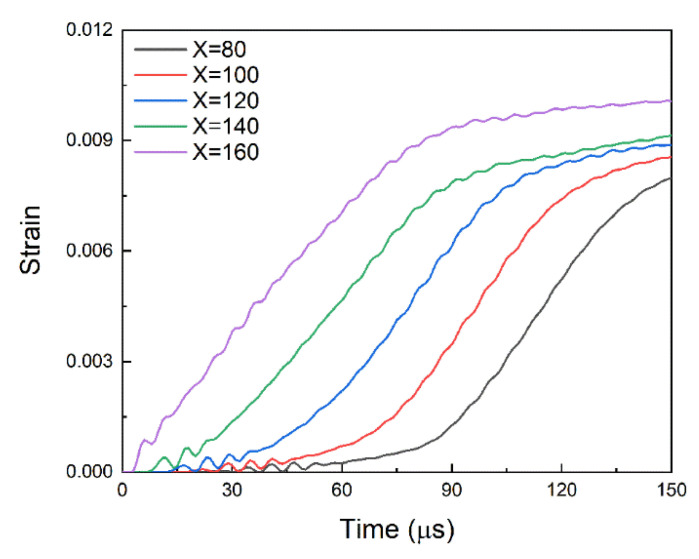
Strain history of FRP under the slip rate of 10 m/s.

The strain rate effects on both FRP stiffness and brick strength were taken into account. The bond–slip relationships were calculated with different slip rates. The slip rate here is the rate of local slip at the interface, which is equivalent to the loading rate at the loading end. For easy parameter conversion, the slip rate is given in m/s. The settings and calculation results under different slip rates are shown in Table 6, where the slip rate $\dot{s}$ ranges from 1.67 × 10^−6^ m/s to 10 m/s. As shown in Figure 22, the strain history of FRP under the slip rate of 10 m/s shows that the slope of the linearly rising section of the FRP strain at different locations is almost identical. In this study, the slope of the linearly rising section at X = 160 mm was taken as the strain rate and the corresponding strain rate $\dot{\varepsilon }$ ranges from 1.96 × 10^−5^ s^–1^ to 118.57 s^–1^.

The relationship between the slip rate and the strain rate is initially obtained from the single-lap shear tests. Then, the material parameters for different strain rates and the corresponding slip rates were set in the numerical simulations. The numerical results under different slip rates further validate the relationship between the slip rate and the strain rate, which was expressed by Equation (13).

(13) $$\log_{10}\dot{\varepsilon }=\log_{10}\dot{s}+1.0687$$

**Figure 23:**
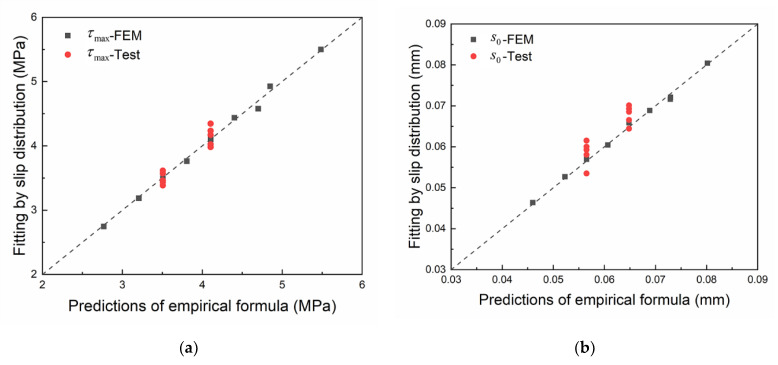
Verification of empirical formulas: (**a**) the maximum bond stress; (**b**) the slip at the maximum bond stress.

The dynamic FRP stiffness and dynamic brick strength at a given slip rate can be calculated by combining Equations (9), (10) and (13). Then, the bond–slip parameter at a given slip rate can be calculated by substituting the dynamic FRP stiffness and dynamic brick strength into the empirical formulas (11) and (12). The maximum bond stress and the corresponding slip calculated by the empirical formulas were compared with the test and numerical results. As shown in Figure 23, the prediction results of the empirical formulas are in good agreement with the test and numerical results, indicating the reliability of the empirical formulas for calculating the bond–slip relationship at the FRP-to-brick interface under dynamic loading.

#### 4.3.2. Analysis and Discussion

**Figure 24:**
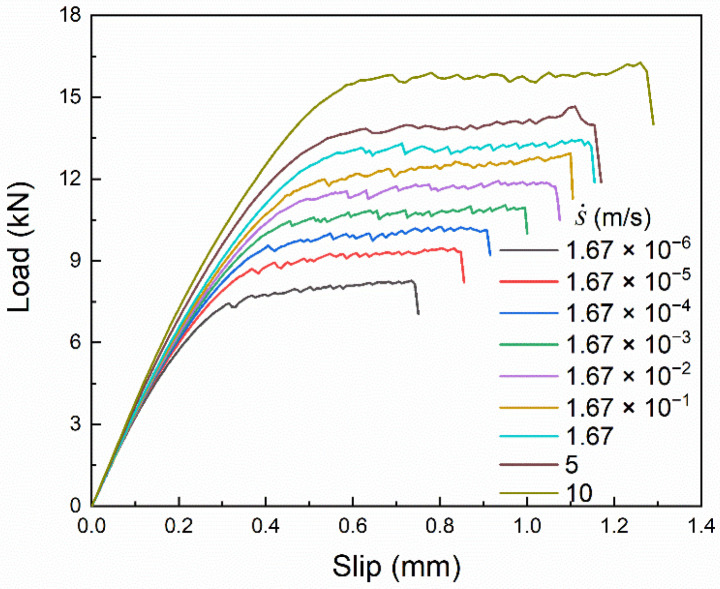
Load–slip curves under different slip rates.

**Figure 25:**
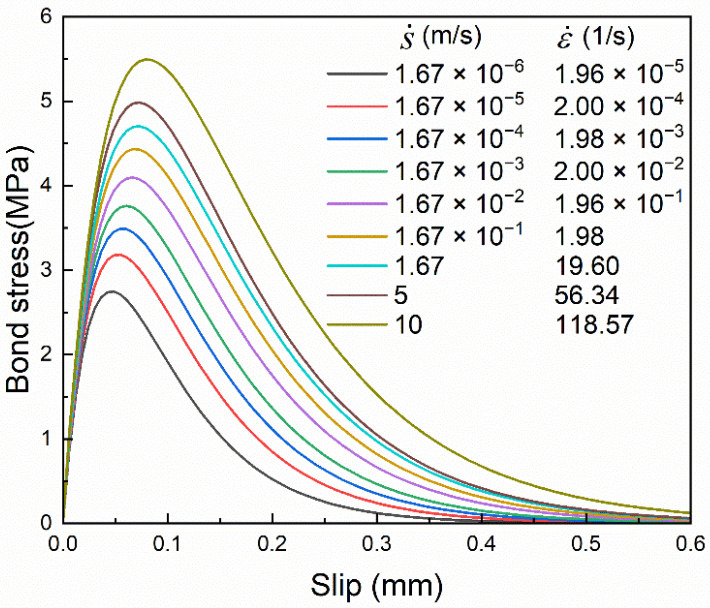
Bond–slip curves under different slip rates.

The load–slip curves and bond–slip curves under different slip rates are shown in Figure 24 and Figure 25, respectively. The variations of the curves under different slip rates are basically similar to those with different brick strength; that is, the curve shape remains unchanged and the amplitude increases. This indicates that the influence of the slip rate on the bond–slip relationship is dominated by the brick strength. The specific reasons can be analyzed with the empirical formulas and the dynamic enhancement law of material performance.

On one hand, the comparison between Figure 18b and Figure 21b shows that the maximum bond stress and the corresponding slip are more sensitive to brick strength than FRP stiffness, and the interfacial fracture energy is only affected by brick strength. On the other hand, the dynamic enhancement effect on brick strength is always greater than FRP stiffness, as shown in Table 4, where the $DIF_{f_{c}}$ is 1.735 at the strain rate of 19.6 s^−1^, while the $DIF_{E_{f}t_{f}}$ is only 1.028.

**Figure 26:**
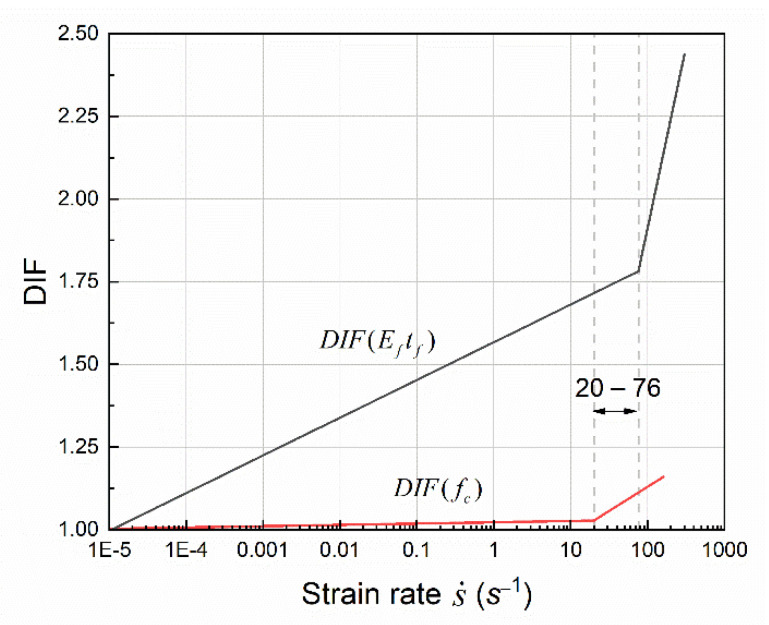
The dynamic enhancement laws of FRP stiffness and brick strength.

Noteworthy, the dynamic enhancement laws of FRP stiffness and brick strength in Equations (9) and (10) are shown in Figure 26. In the strain rate range of 20–76 s^−1^ (the slip rate is 1.71–6.49 m/s), the strain rate sensitivity (slope of the curve) of FRP stiffness is slightly stronger than that of brick strength. In this case, the bond–slip relationship is clearly influenced by FRP stiffness, which is the main reason for the shape variation of the bond–slip curve under the slip rate of 5 m/s. However, as the strain rate increases further and becomes greater than 76 s^−1^ (the slip rate is greater than 6.49 m/s), the strain rate sensitivity of brick strength increases significantly and becomes much greater than the strain rate sensitivity of FRP stiffness, as shown in Figure 26. The dynamic enhancement effect on brick strength is therefore more pronounced and again dominates the variations in the bond–slip relationship under dynamic loading, as can be seen from the bond–slip curve under the slip rate of 10 m/s.

In summary, the variations of the bond–slip relationship under dynamic loading are mainly caused by the dynamic enhancement of brick strength. In other words, the amplitude of the bond–slip curve increases with increasing slip rate, and the shape of the curve almost remains unchanged. However, the bond–slip relationship is also clearly influenced by FRP stiffness especially within a specific slip rate range, where the strain rate sensitivity of FRP stiffness is stronger than that of brick strength.

## 5. Conclusions

In this paper, the effects of FRP stiffness, brick strength and slip rate on the bond–slip relationship at FRP-to-brick interfaces are numerically investigated based on the single-lap shear tests with two different loading rates. The conclusions are as follow:

1. The numerical model based on the plastic-damage constitutive and the strain rate effects on material performance can simulate the FRP-to-brick interface behavior under different loading rates, and the numerical results of the bond–slip relationship are in good agreement with the test results.
2. FRP stiffness mainly influences the shape of the bond–slip curve. As the maximum bond stress increases with increasing FRP stiffness, the corresponding slip at the maximum bond stress decreases and the interfacial fracture energy remains constant.
3. Brick strength mainly influences the amplitude of the bond–slip curve. Both the maximum bond stress and the corresponding slip increase as the brick strength increases, and the shape of the bond–slip curve remains almost unchanged; thus, the interfacial fracture energy increases significantly.
4. The variations of the bond–slip relationship under dynamic loading are mainly a consequence of the dynamic enhancement of brick strength, so the magnitude of the bond–slip curve changes significantly. However, the dynamic bond–slip relationship is also influenced by FRP stiffness especially within a specific slip rate range, where the strain rate sensitivity of FRP stiffness is stronger than that of brick strength.
5. The empirical formulas considering dynamic FRP stiffness and dynamic brick strength can be used to predict the bond–slip relationship at the FRP-to-brick interface under dynamic loading, and its prediction results are in good agreement with the test and numerical results.

## Figures and Tables

**Table 1:** Properties of the materials.

Material	Type	Tensile/Compression Strength (MPa)	Young’s Modulus (GPa)	Failure Strain (%)	Nominal Thickness (mm)
Carbon fabric	UT70-30G	+4000/—	253	1.7	0.167
Epoxy adhesive	HM-180C3P	+38/—	2.4	1.5	—
Clay brick	MU15	+2.85/−11.4	7.6	—	—

**Table 2:** The bond–slip parameters from the tests.

Specimens	Loading Rate (mm/min)	*α* (mm)	*β* (mm)	$s_{0}$ (mm)	$\tau_{\max}$ (MPa)	$G_{f}$ (N/mm)
A1	10	0.0851	15.92	0.0590	3.551	0.6044
A2	10	0.0803	16.58	0.0557	3.090	0.4963
A3	10	0.0949	16.06	0.0658	3.892	0.7387
A4	10	0.0855	16.17	0.0593	3.461	0.5919
A5	10	0.0796	15.90	0.0552	3.330	0.5302
Average	10	0.0851	16.13	0.0590	3.465	0.5923
B1	1000	0.1058	15.63	0.0733	4.581	0.9693
B2	1000	0.0989	15.84	0.0686	4.168	0.8245
B3	1000	0.0971	15.76	0.0673	4.138	0.8036
B4	1000	0.0992	16.02	0.0688	4.087	0.8108
B5	1000	0.0921	15.90	0.0638	3.853	0.7097
Average	1000	0.0986	15.83	0.0684	4.165	0.8236

**Table 3:** Plastic parameters of clay brick.

ψ (°)	*ξ*	*f*_b0_/*f*_c0_	*K*	Viscosity Parameter
38	0.1	1.16	0.6667	1 × 10^−5^

**Table 4:** Calculation results with different FRP stiffness.

Specimens	$E_{f}$ (MPa)	$t_{f}$ (mm)	*α* (mm)	*β* (mm)	$\bar{E_{f}t_{f}}$	$\bar{\alpha }$	$\bar{\beta }$	${\bar{\tau }}_{\max}$	${\bar{s}}_{0}$	${\bar{G}}_{f}$
CFRP-1ply	253,388	0.167	0.0669	16.05	1.00	1.00	1.00	1.000	1.000	1.000
CFRP-1ply-1.2	304,066	0.167	0.0650	16.84	1.20	0.97	1.05	1.059	0.972	1.029
CFRP-1ply-1.4	354,743	0.167	0.0633	17.92	1.40	0.95	1.12	1.063	0.946	1.005
CFRP-1ply-1.6	405,421	0.167	0.0617	18.56	1.60	0.92	1.16	1.105	0.923	1.019
CFRP-1ply-1.8	456,098	0.167	0.0605	19.47	1.80	0.91	1.21	1.107	0.905	1.002
CFRP-2ply	253,388	0.334	0.0596	20.31	2.00	0.89	1.27	1.114	0.892	0.993
CFRP-3ply	253,388	0.501	0.0568	23.33	3.00	0.85	1.45	1.206	0.849	1.025
GFRP-1ply	84,251	0.120	0.0833	9.59	0.24	1.25	0.60	0.835	1.246	1.040
GFRP-2ply	84,251	0.240	0.0757	12.57	0.48	1.13	0.78	0.882	1.132	0.999
GFRP-3ply	84,251	0.360	0.0730	14.53	0.72	1.09	0.91	0.955	1.092	1.043
BFRP-1ply	88,397	0.140	0.0821	10.47	0.29	1.23	0.65	0.843	1.228	1.036
BFRP-2ply	88,397	0.280	0.0743	13.28	0.58	1.11	0.83	0.950	1.111	1.055
BFRP-3ply	88,397	0.420	0.0669	15.17	0.88	1.00	0.95	0.982	1.000	0.982

**Table 5:** Calculation results under different brick strength.

$f_{c}$ (MPa)	*α* (mm)	*β* (mm)	${\bar{f}}_{c}$	$\bar{\alpha }$	$\bar{\beta }$	${\bar{\tau }}_{\max}$	${\bar{s}}_{0}$	${\bar{G}}_{f}$
11.40	0.0669	16.05	1.00	1.00	1.00	1.000	1.000	1.000
13.29	0.0760	16.07	1.17	1.14	1.00	1.134	1.136	1.289
14.59	0.0821	15.77	1.28	1.23	0.98	1.273	1.228	1.563
15.88	0.0833	15.83	1.39	1.25	0.99	1.281	1.245	1.595
17.18	0.0947	15.70	1.51	1.42	0.98	1.480	1.415	2.096
18.48	0.1001	15.61	1.62	1.50	0.97	1.583	1.496	2.368
19.78	0.1068	15.55	1.74	1.60	0.97	1.701	1.597	2.716
21.81	0.1157	15.31	1.91	1.73	0.95	1.902	1.730	3.290
25.57	0.1317	15.25	2.24	1.97	0.95	2.182	1.969	4.295

**Table 6:** Calculation results under different slip rates.

$\dot{s}$ (m/s)	$\dot{\varepsilon }$ (1/s)	$DIF_{E_{f}t_{f}}$	$DIF_{f_{c}}$	*α* (mm)	*β* (mm)	$\bar{\alpha }$	$\bar{\beta }$	${\bar{\tau }}_{\max}$	${\bar{s}}_{0}$	${\bar{G}}_{f}$
1.67 × 10^−6^	1.96 × 10^−5^	1.000	1.000	0.0669	16.052	1.000	1.000	1.000	1.000	1.000
1.67 × 10^−5^	2.00 × 10^−4^	1.009	1.165	0.0760	15.889	1.136	0.990	1.160	1.136	1.318
1.67 × 10^−4^	1.98 × 10^−3^	1.011	1.279	0.0821	15.766	1.228	0.982	1.273	1.228	1.563
1.67 × 10^−3^	2.00 × 10^−2^	1.016	1.393	0.0872	15.659	1.303	0.976	1.370	1.303	1.785
1.67 × 10^−2^	1.96 × 10^−1^	1.020	1.507	0.0950	15.659	1.421	0.976	1.493	1.421	2.121
1.67 × 10^−1^	1.98	1.024	1.621	0.0993	15.390	1.485	0.959	1.615	1.485	2.399
1.67	19.60	1.028	1.735	0.1033	15.452	1.544	0.963	1.666	1.544	2.574
5.00	56.34	1.093	1.766	0.1040	15.633	1.555	0.974	1.815	1.555	2.946
10.00	118.57	1.141	1.994	0.1160	15.959	1.735	0.994	2.002	1.735	3.473

## Data Availability

The raw/processed data required to reproduce these findings cannot be shared at this time as the data also form part of an ongoing study.
